# From Local Structure to Overall Performance: An Overview on the Design of an Acoustic Coating

**DOI:** 10.3390/ma12162509

**Published:** 2019-08-07

**Authors:** Hongbai Bai, Zhiqiang Zhan, Jinchun Liu, Zhiying Ren

**Affiliations:** 1 College of Mechanical Engineering and Automation, Fuzhou University, Fuzhou 350116, China; 2 Engineering Research Center for Metal Ruber, Fuzhou University, Fuzhou 350116, China

**Keywords:** acoustic stealth, acoustic coating, passive sound absorption, active sound absorption, acoustic characteristics of a submarine, finite element method (FEM)

## Abstract

Based on the requirements of underwater acoustic stealth, the classification and research background of acoustic coatings are introduced herein. The research significance of acoustic coatings is expounded from the perspective of both the military and civilian use. A brief overview of the conventional design process of acoustic coatings is presented, which describes the substrates used in different countries. Aimed at the local design of acoustic coatings, research progress on passive and semi-active/active sound absorption structure is summarized. Focused on the passive acoustic coatings; acoustic cavity design and optimization, acoustic performance of acoustic coatings with rigid inclusions or scatterers, and acoustic coatings with a hybrid structure are discussed. Moreover, an overview of the overall design of acoustic coatings based on the sound field characteristics of the submarine is also presented. Finally, the shortcomings of the research are discussed, breakthroughs in acoustic coating design research are forecast, and the key technical issues to be solved are highlighted.

## 1. Introduction

**Figure 1:**
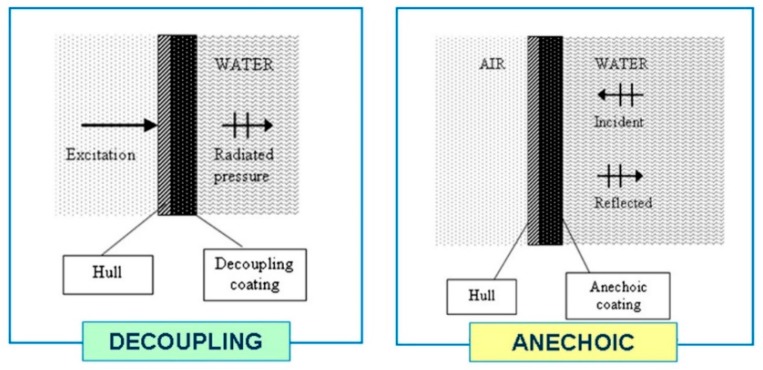
Two main types of acoustic coating [9].

As acoustic stealth equipment widely used in submarines, the acoustic coating can absorb the sound waves emitted by the active sonar. It can also suppress the vibration of the hull and isolate the noise inside the boat. Therefore, the acoustic coating becomes the only key component on the submarine that can effectively counter active enemy sonar and passive sonar detection. Based on the sound field characteristics of the submarine, namely, radiation noise characteristics, self-noise characteristics, and target strength characteristics [1], acoustic coatings can be roughly divided into two types according to function: sound insulation decoupling tiles and anechoic tiles, as shown in Figure 1. The main function of decoupling tiles is to reduce the radiation noise and self-noise of the submarine. In contrast, anechoic tiles reduce the target strength characteristics of the submarine and the reflection of active sonar sound waves. Moreover, some acoustic coatings actually have both the functions mentioned above.

In practice, properties of acoustic materials can change under hydrostatic pressure conditions (such as the shape of the cavity, modulus of materials, etc.), which affects the sound absorption performance of the materials. With the development of low-frequency sonar, high-efficiency sound absorption of underwater acoustic stealth materials with finite thickness under low frequency (generally considered to be below 1 kHz) and wide frequency conditions has become a hot topic in current research [2]. In the military field, the acoustic coating is one of the effective means to improve the sound stealth performance of submarine. The “quiet submarine” with an acoustic coating has become an important direction for the development of modern submarines, which has practical significance for the development of naval equipment and the construction of national defense military equipment.

In the civilian field, acoustic coating generally refers to various functional materials used underwater. In civil water-sound systems, involving seabed resource exploration [3], seabed mapping [4], fish stock information detection [5], and remote sensing [6], acoustic coatings can absorb unnecessary sound waves in the marine environment to improve the accuracy of positioning, searching, and the communication capability of the equipment. In addition, acoustic coatings play an important role in improving the marine environment and reducing noise pollution [7]. Moreover, the acoustic coatings should be used to cover the muffler pool during the measurement or calibration of underwater acoustic equipment and other hydroacoustic tests [8].

To acquire a better understanding of the design of acoustic coatings, in this study a brief overview of the conventional design process for acoustic coatings is provided first, and then the local structure design of acoustic coatings was introduced (especially for the design of passive acoustic coatings). An overview of the overall performance of acoustic coatings was also outlined. Finally, the shortcomings of the research were discussed, followed by the development of a new prospect.

## 2. Conventional Design Process for Acoustic Coatings

**Figure 2:**
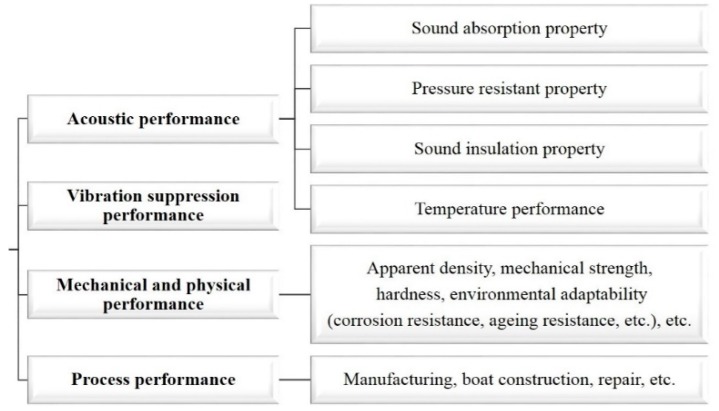
Performance requirements for an acoustic coating [1].

**Figure 3:**
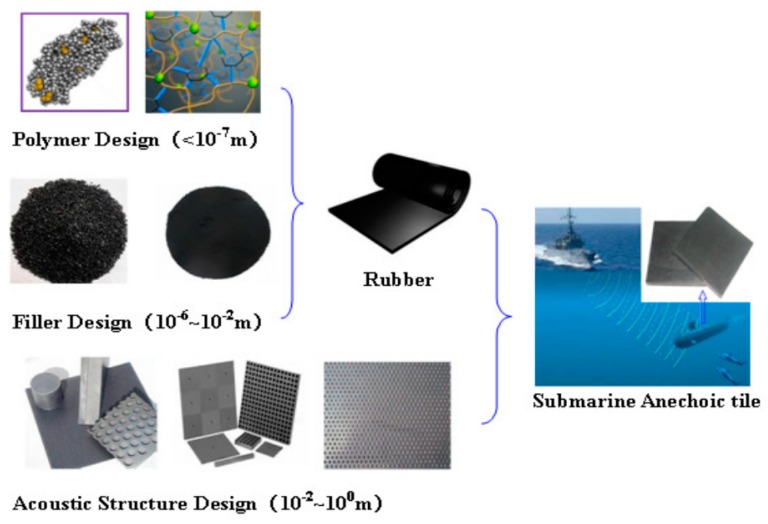
Conventional design process of acoustic coatings [10].

The design process of the acoustic coating was developed around the performance requirements presented in Figure 2, starting from small-scale design to large-scale design, as shown in Figure 3. The conventional acoustic coating design process begins with polymer and filler design while carrying out the local acoustic structure design, and finally, the overall performance design (including the hull). The formula design is the most important step in the process, which mainly involves viscoelastic polymer materials, such as butyl rubber, styrene-butadiene rubber, polyurethane, polysulfide rubber, polybutylene rubber, neoprene, fiber-reinforced polymer, polyurethane/epoxy copolymer, silicone rubber, etc., as substrates. Different countries use different substrate materials in acoustic coatings, as presented in Table 1.

## 3. Local Design of Acoustic Coatings

Local design of acoustic coatings mainly refers to designing of local acoustic structure. Acoustic coatings are usually laid on the hull piece by piece; therefore, design of a single-piece acoustic coating (or unit structure) can also be defined as local design. Local design consists of passive and semi-active/active acoustic coatings’ design (including research on pressure resistance, sound insulation, etc.). Passive sound absorption design involves optimization of acoustic cavities, acoustic performance of acoustic coatings with rigid inclusions or scatterers, and hybrid structure acoustic coatings. In contrast, semi-active or active sound absorption is achieved by semi-active or active control of sound waves.

### 3.1. Acoustic Cavity Design and Optimization on Passive Acoustic Coatings

The origin of the world’s first passive acoustic structure dates back to the end of World War II when the concept of German “Alberich”-type acoustic coating was introduced. The historical synthetic rubber coatings installed on German submarines were designed with lattices of resonant cavities, which had sound-absorbing properties [12]. With the increasing complexity of acoustic coatings, analytical methods are difficult to use for calculations of complex structures. Since the 1990s, Hennion [13] and Easwaran [14] began to study acoustic coatings by the finite element method (FEM). Nowadays, with the development of numerical simulation software (for example, ANSYS, COMSOL, etc.), the FEM can not only optimize the traditional structure but also facilitate the research of new material structure.

**Figure 4:**
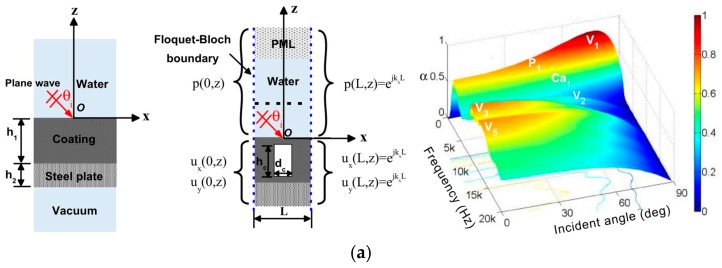

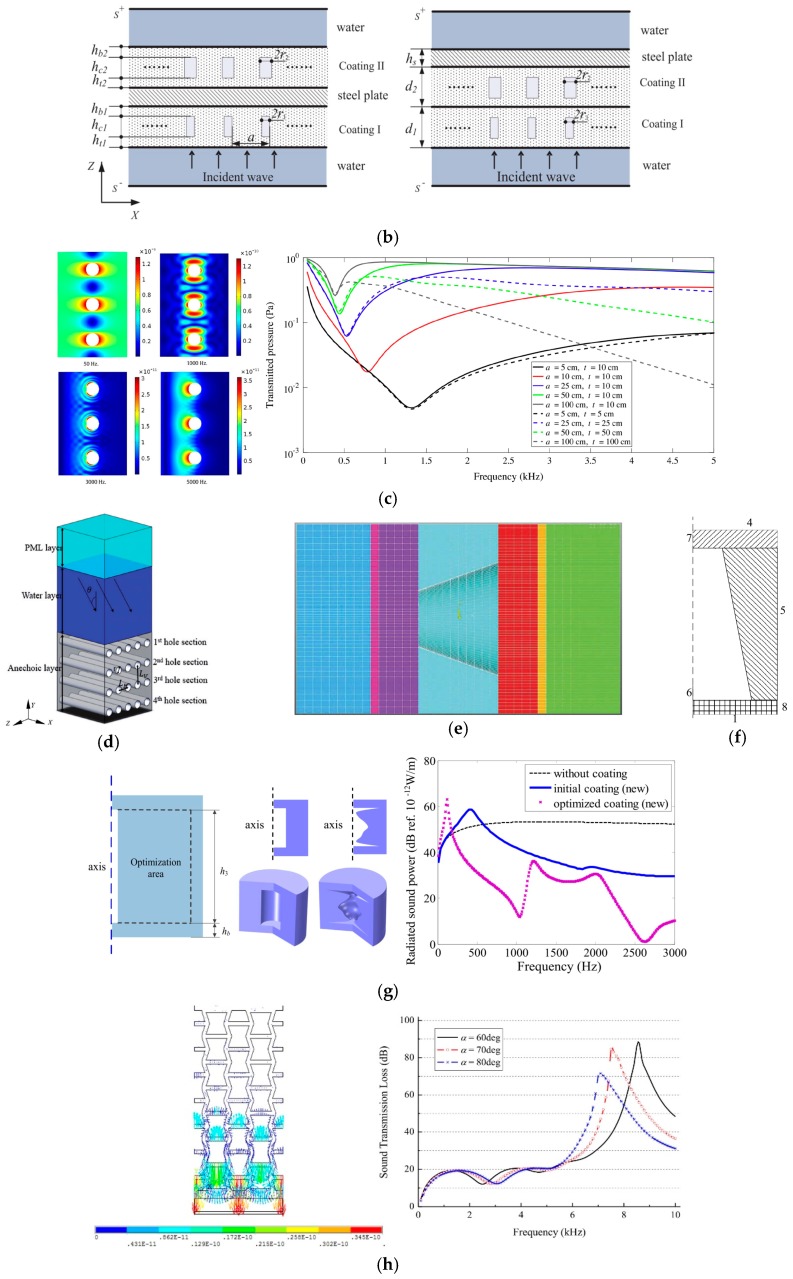
Design and optimization of the acoustic cavity. (**a**) Two-dimensional analytical model, periodic numerical model, and sound absorption coefficients of the Alberich anechoic coating [15]. (**b**) Coatings on both surfaces and the outer surface of a steel plate and their absorption coefficients [16]. (**c**) Deformation of the polydimethylsiloxane (PDMS) medium and transmitted pressure obtained analytically [18]. (**d**) Schematic of the finite element method (FEM) model [19]. (**e**) Cross-section of the absorbent coating, including a cone frustum hole [20]. (**f**) Two-dimensional axisymmetric model [21]. (**g**) Sketch of the optimization area for the cavity, initial cavity, optimized cavity, and radiated sound power associated with different coatings attached to an infinite plate [22]. (**h**) Sound transmission loss and displacement contour of the negative Poisson’s ratio honeycomb-hole coatings [23].

The study of oblique incidence provides a more practical and comprehensive significance for characterizing the acoustic performance of the Alberich-type acoustic coating; therefore, sound absorption performance of the steel-backed acoustic coating was studied when the acoustic wave was obliquely incident. By using numerical calculation methods, the sound absorption coefficient of the Alberich-type acoustic coating was calculated, and the mechanism of sound absorption was studied in terms of the structural displacement vector and deformation, as presented in Figure 4a. Notably, there is a wide and strong silencer region at larger angles of incidence and lower frequency. However, at higher frequencies, the sound absorption coefficient decreases with the increase in the angle of incidence [15].

Most of the current researches focus on acoustic coatings laid on the surface of rigid steel plates; however, different acoustic coatings laid on the surface of water-immersed steel sheets have rarely been investigated. Therefore, differential evolution algorithms combined with FEM were used for optimizing two types of Alberich acoustic coatings. The two resonance modes of acoustic coatings were different; therefore, we could obtain good absorption properties through acoustic coupling between two acoustic coatings and the cooperation with the damping rubber [16], as shown in Figure 4b.

Acoustic and physical characteristics of acoustic coatings can be modified by adding various components. By redesigning materials and structures of the Alberich-type acoustic coating, the tapered bore structure led to the enhancement in its low-frequency performance in the use of underwater testing and calibration cells [17].

In the aspect of the establishment and calculation of an analysis model for acoustic coatings, an analytical model was established based on the homogenization theory, which has higher computational efficiency. The results shown in Figure 4c indicate that strong coupling of cavity resonance leads to broadband attenuation of the sound [18]. According to the accurate requirements of viscoelastic dynamic parameters, such as complex elastic modulus and Poisson’s ratio, a new parameter identification method was proposed based on the measured reflection coefficient. Acoustic properties of acoustic coating with horizontally distributed cylindrical holes were also studied, as shown in Figure 4d. The results showed that the smaller the horizontal spacing between two adjacent holes, the better the low-frequency sound absorption effect [19].

Most literatures have studied the optimization of structure and material on single-layer acoustic coatings; however, acoustic coatings with a multilayer structure have not been extensively studied. A program based on finite element simulation was developed in ANSYS. Through the comparison between a cone frustum hole and a cylindrical hole, the frustum hole (as shown in Figure 4e) was found to have a large transmission coefficient [20]. A finite element model with the frustum cavity (as shown in Figure 4f) was constructed by using COMSOL software. The results showed the superior absorption properties of the multilayered structure, and the sound absorption performance of the frustum cavity was better than that of the cylindrical and ellipsoidal cavities [21].

The development of finite element simulation technology is of great significance, which aids in the design of complex acoustic cavities. To improve the decoupling ability of acoustic coating, a complex shape of the cavity was designed by optimization, as shown in Figure 4g. Furthermore, an equivalent fluid model was established for processing acoustic coatings with periodically distributed and axisymmetric cavities [22]. The sound insulation performance of two types of honeycomb acoustic coatings was studied, and it was concluded that the negative Poisson’s one has better sound insulation performance at a certain frequency [23], as shown in Figure 4h. The effectiveness of the use of small air Helmholtz resonator structure was studied, and the echo suppression results showed that the resonator has a broad application prospect in achieving underwater stealth [24].

With the deepening of research in this area, pressure resistance performance of acoustic coatings has also received increasing attention. Under hydrostatic pressure, acoustic properties of acoustic coatings change. On the one hand, dynamic mechanical properties of materials change with pressure, which leads to the change in parameters of acoustic coating materials. On the other hand, when the acoustic coating is subjected to hydrostatic pressure, the cavity is deformed such that the structural parameters of acoustic coatings change.

In terms of the influence of hydrostatic pressure on sound absorption performance, a spherical cavity acoustic coating unit model was built by using COMSOL software, which was based on the two-dimensional (2D) theory of sound waves under normal incidence. A coupled model of acoustic performance calculation based on the compression deformation of the cavity was obtained, and the influence of the internal pressure of the cavity was considered. With increasing hydrostatic pressure, the peak frequency was toward the high frequency and became wider, absorption effect became more significant for overall degradation, and internal air pressure reduced the cavity deformation and improved the low-frequency sound absorption effect [25].

**Figure 5:**
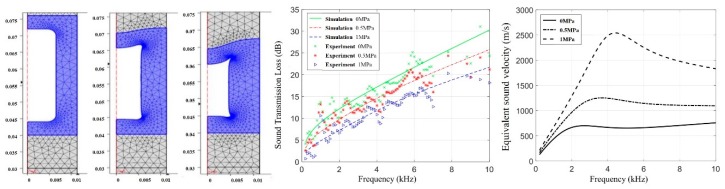
Acoustic properties of acoustic coatings under hydrostatic pressure. Deformed shape of the unit cell, transmission loss, and the equivalent sound velocity of the sound insulation layer under different static pressure [26].

For the problem of the sound insulation effect associated with sound insulation decoupling material under hydrostatic pressure, the influence of hydrostatic pressure on the insulation performance of acoustic coating was analyzed, as shown in Figure 5. With the increase in the hydrostatic pressure, the equivalent sound velocity of the material increased, and the sound insulation performance of acoustic coating decreased [26]. Therefore, deformation resistance of the material under pressure can be improved by adding a rigid confinement structural unit inside the acoustic cavity of the sound insulation decoupling materials [27].

### 3.2. Acoustic Properties of Passive Acoustic Coatings with Rigid Inclusions or Scatterers

Structural design of the acoustic cavity better solves the problem of low-frequency sound absorption, but it also needs to consider its pressure resistance performance. A United States Patent discloses a composite material comprising a spherical shell inclusion for a subsea platform, which exhibited a strong static stiffness [28]. This provides an idea for the study of the relationship between acoustic and pressure properties of acoustic coatings, i.e., enhancement of pressure resistance by adding rigid inclusions or scatterers. Traditional underwater acoustic stealth materials are limited by the mass density law. Larger sized of materials are required to increase the acoustic wave propagation path for improving the energy dissipation of low-frequency sound waves. Of note, local resonant acoustic metamaterials can achieve long-wavelength sound waves’ control at a small scale, which provides ideas for solving low-frequency sound absorption problems [8].

**Figure 6:**
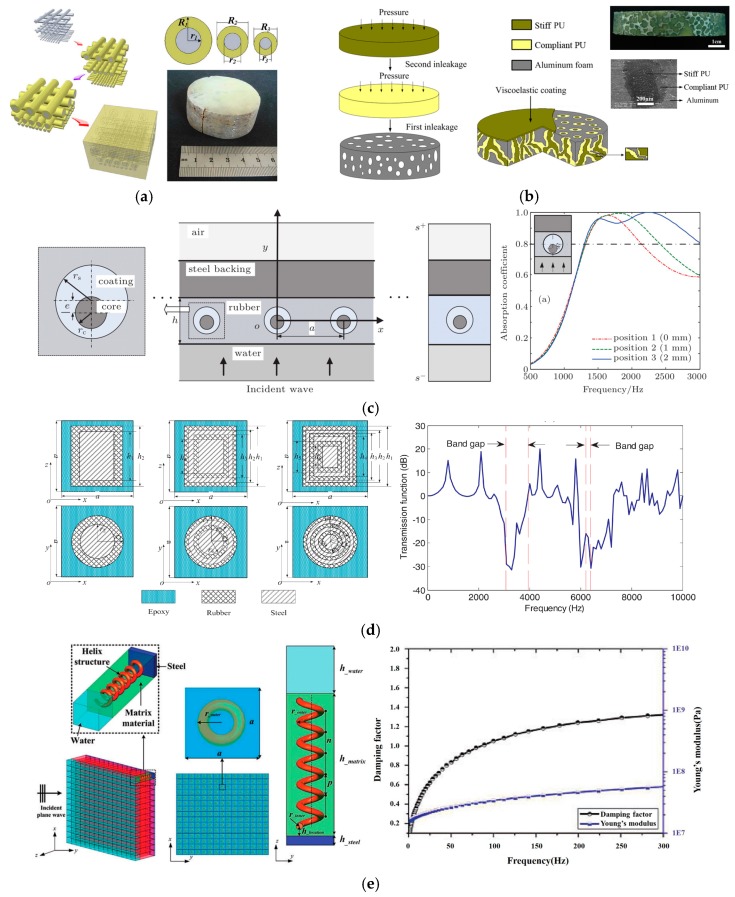
Acoustic properties of acoustic coatings with rigid inclusions or scatterers. (**a**) Schematic of synthesis route and optical image of locally resonant phononic woodpile [31]. (**b**) Schematic of synthesis, the structure of phononic glass, optical and SEM images of a typical phononic glass sample [32]. (**c**) Cross-section of one locally resonant scatterer unit, absorption structure with steel backing, one Bloch unit cell, and comparison among the absorptances for three core positions [35]. (**d**) Conventional local resonance acoustic metamaterials (LRAMs), double-layered cylindrical scatterers, three-layered cylindrical scatterers, and band diagram of the LRAMs with three-layered locally resonant scatterers [36]. (**e**) Three-dimensional geometric dimensions and experimental results of dynamic mechanical parameters of viscoelastic damping rubber [37].

In 2000, Liu et al. proposed the concept of locally resonant sonic metamaterials. A lead ball coated with a layer of soft silicone rubber was embedded in the hard epoxy resin matrix to form a three-dimensional lattice structure. The acoustic band gap was generated by resonance, thereby achieving the purpose of absorbing sound waves and dissipating energy [29]. Comparison of sound absorption properties between spherical and cylindrical local resonance structures indicated that a cylindrical one can further improve the low-frequency sound absorption performance [30]. Use of a locally resonant phononic woodpile structure (as shown in Figure 6a) provided a new approach to solve the associated weight loss problem [31]. Figure 6b shows a phononic glass with an interpenetrating network structure, exhibiting dual characteristics of high mechanical strength and excellent underwater sound absorption capacity [32].

For a more realistic understanding, the influence of frequency-dependent material parameters on the performance of local resonance acoustic metamaterials (LRAMs), a generalized Maxwell model was used to simulate the behavior of multi-polymer materials. Studies have shown that the parameters of the rubber coating material vary with the frequency and influence the wave attenuation effect in the frequency range around the band gap [33]. The acoustic performance of locally resonant phononic crystals consisting of periodic steel cylinders in a viscoelastic rubber medium was studied analytically and numerically, and the approach may be useful for customized designs of acoustic metamaterials, such as rapid prototyping and trend analysis, in underwater applications [34].

Current research focuses on symmetric resonant cavities, such as spherical resonators or coaxial cylindrical resonant cavities. However, characteristics of non-symmetric resonators, such as non-concentric resonators or non-coaxial resonators, have been less studied. Thus, FEM was used to study the absorption characteristics of a viscoelastic plate periodically embedded in an infinitely long non-coaxial cylindrical local scatterer, as shown in Figure 6c. The results indicated the existence of two typical resonance modes: One is the overall resonance mode caused by the steel back, in which the core position had little effect on it. The other is the core resonance mode caused by the local resonance scatterer. Furthermore, with the increase in the core eccentricity, the core resonance mode moved toward the high-frequency direction [35].

To overcome the shortcomings of the narrow range of the effective sound-absorption frequency of local resonance phononic crystals, a novel multilayer local resonance acoustic metamaterial was proposed, in which a multilayer local resonance scatterer was embedded into the matrix structure, as shown in Figure 6d. Coupling resonance expanded the band gap and enhanced the sound absorption performance of metamaterials [36].

Since the existing technology cannot realize effective absorption of subwavelength structure, an underwater element structure was proposed to achieve low-frequency broadband sound absorption below 1000 Hz, which was based on the structural combination design of common viscoelastic damping materials and metal materials, as shown in Figure 6e. Physical mechanisms of acoustic coating included waveform transformation, multiple scattering, etc. Different geometries and materials of the helical structure aided in the adjustment of the sound absorption properties of materials [37].

### 3.3. Passive Acoustic Coatings with Hybrid Structure

**Figure 7:**
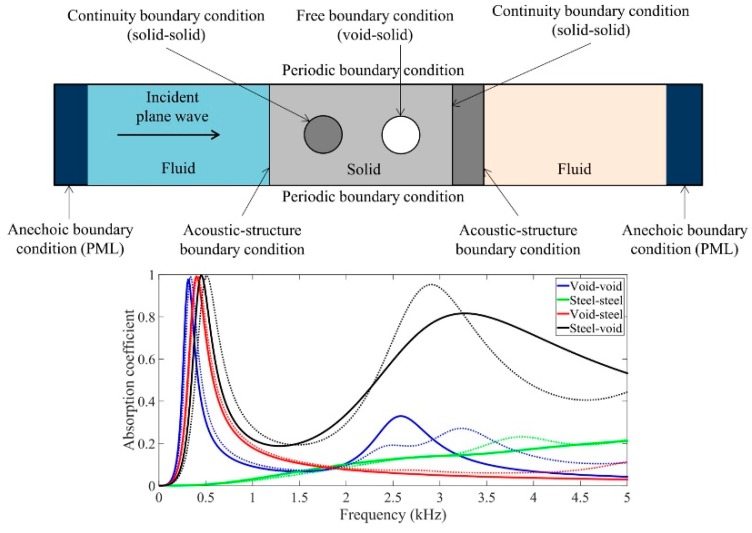
Acoustic coatings with hybrid structure. Schematic diagram of the numerical model and effect of steel backing plate on the sound absorption coefficient of a phononic crystal [39].

An acoustic coating with a hybrid structure takes sound absorption and pressure resistance performance into account, which incorporates other rigid materials while retaining the acoustic cavity. Sound absorption performance of a composite structure combining rubber material containing a cylindrical cavity and porous metal material was studied. The results revealed that the addition of porous metal material could improve the low-frequency sound absorption capability [38]. For acoustic coating containing both cavities and hard scatterers, the equivalent medium theory was used to analyze and numerically calculate underwater sound absorption characteristics of phononic crystals in soft rubber media. A finite element model for the periodic distribution of cavities and hard inclusions in the rubber media was established, as shown in Figure 7. It has been found that acoustic coating consisting of a layer of hard inclusions plus a small cavity in the direction of sound propagation exhibits a high sound absorption capacity over a wide frequency range [39].

### 3.4. Design on Semi-Active/Active Acoustic Coatings

Passive sound absorption technology relies on the structural design of acoustic coatings and material modification; however, its sound absorption mechanism is limited, and control of low frequency and wide frequency sound waves is not good. Therefore, a semi-active/active control method for sound waves was proposed.

**Figure 8:**
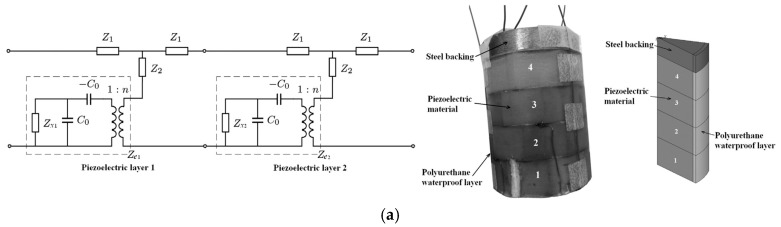

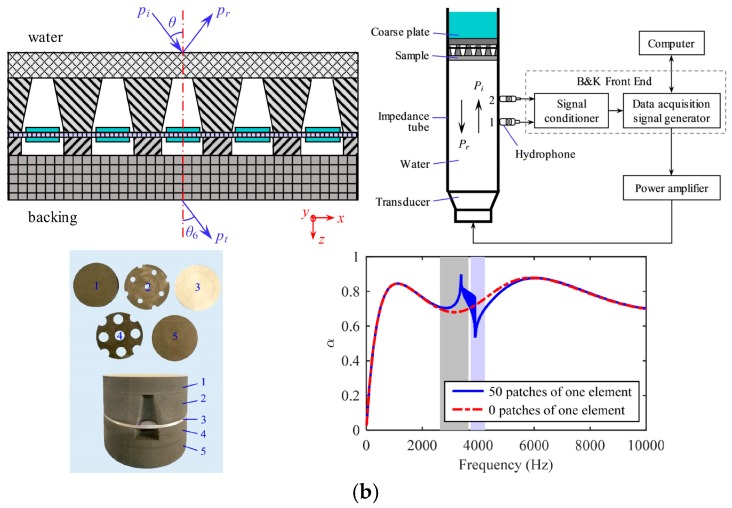
Design on semi-active acoustic coatings. (**a**) Mason equivalent circuit of two piezoelectric layers, experimental sample and finite element model with polyurethane waterproof layer [41]. (**b**) Schematic diagram of the composite coating, test schematic of a hydroacoustic impedance tube, test sample, and comparison between theoretical predictions of the coating [42].

Piezoelectric composites have been widely used in semi-active acoustic coatings. Through internal loss, part of the acoustic energy can be directly converted into thermal energy; another part of the acoustic energy can be converted into electrical energy due to the piezoelectric effect and then converted into thermal energy by piezoelectric passive control. The effect of rubber damping materials in the piezoelectric parallel sound absorption layer was investigated, and a design for acoustic coating with a multilayer structure (damping rubber, piezoelectric composite and lead zirconate titanate (PZT) ceramic) was proposed, which effectively improved the low-frequency sound absorption coefficient [40]. Combined with parallel impedance under the constraint of the underwater finite frequency sound absorption coefficient, a practical multi-layer piezoelectric acoustic coating sound absorption control method was proposed [41], as shown in Figure 8a. Another design of semi-active compound acoustic coating exhibited periodic sub-wavelength piezoelectric array, which broadened the bandwidth of the first absorption peak [42], as presented in Figure 8b.

**Figure 9:**
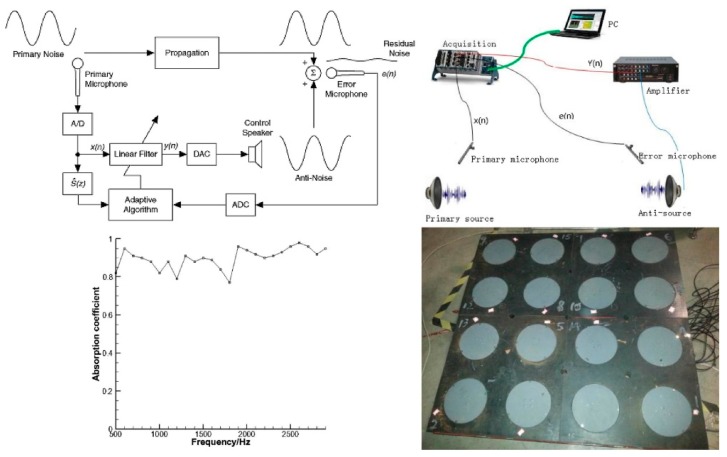
Design on active acoustic coatings. Active control procedure, absorption coefficients at the stable pressure value of 0.96 MPa and an active large plate sample [44].

Active sound absorption controls the secondary sound source to generate sound waves that are opposite to the primary sound source through the principle of coherent cancellation, thus achieving the objective of active sound absorption [11]. A low-frequency echo suppression technique based on active impedance matching was proposed, which employed a tile projector designed to cover a wide area, such as the surface of a ship. Moreover, its low-frequency echo reduction performance was tested [43]. A low-frequency miniaturized active control unit with a thickness of less than 50 mm using giant magnetostrictive material was designed, which was able to achieve significant noise reduction in less than a one-second interval. Even under high pressure, its sound absorption coefficient was far more than 0.8 [44], as shown in Figure 9.

## 4. Overall Design on Acoustic Coatings

**Figure 10:**
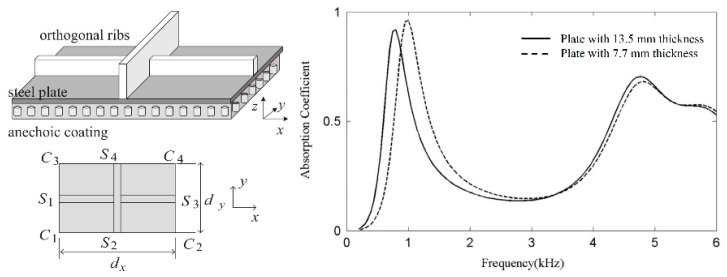
Acoustic radiation of underwater cylindrical shells. Calculating model of a unit cell, its top view, and absorption coefficients of the Alberich anechoic coating backed with different steel plates [47].

In recent years, overall design on acoustic coatings based on the characteristics of the submarine sound field has progressively received more attention, with a focus on acoustic radiation of underwater cylindrical shells, including finite/infinite long cylindrical shells, elastic or viscoelastic single/double shells, stiffened cylindrical shells, etc. The finite element and boundary element model of the submarine section were built, and sound radiation calculations revealed that functionally graded materials exhibited better vibration and noise reduction performance [45]. By matching the velocity method, a method for estimating the equivalent material parameters of acoustic coatings, was proposed, which simplified the problem associated with complex acoustic radiation prediction to the problem of the equivalent homogeneous viscoelastic acoustic coating structure [46]. An equivalent modulus method based on impedance transfer formula was proposed, which reduced the number of calculating nodes. The study indicated that an increase in the mass of backing caused absorption peak to move to low frequency [47], as shown in Figure 10.

**Figure 11:**
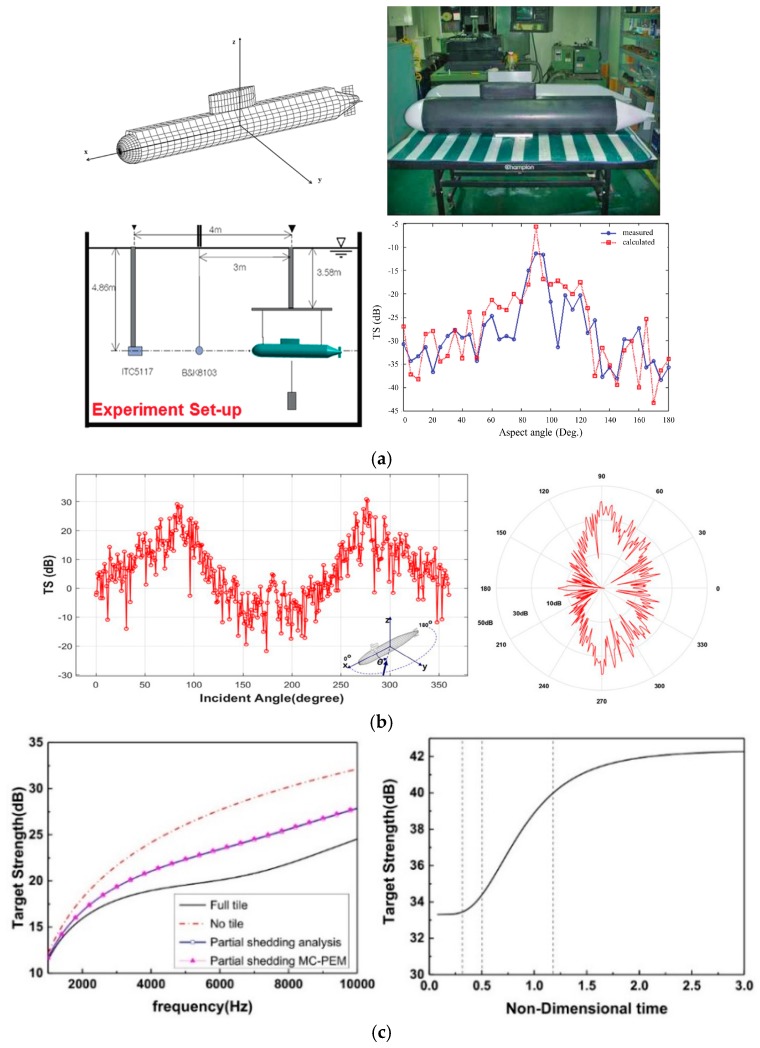
Research on target strength characteristics of the submarine. (**a**) Mesh model, test model, experiment equipment, and comparison of experimental and numerical solutions for target strength (TS) [48]. (**b**) TS of the rectangular and polar coordinate of the submarine model at a frequency of 5 kHz and a distance of 500 m [49]. (**c**) Comparison of the analytical method and Monte Carlo–Plate Element Method (MC-PEM) and the relationship between TS and non-dimensioned time [50].

Other studies were on the target strength characteristics of the submarine. A high-frequency analysis program based on Kirchhoff approximation was developed for the prediction of submarine target strength (TS) [48], as presented in Figure 11a. Figure 11b exhibits the analysis of underwater vehicle model based on the established near-field equation, and the influence of parameters, such as distance and frequency, on the model was researched [49]. Based on Monte Carlo–Plate Element Method (MC-PEM), the relationship between target strength and shedding rate of the cylindrical shell was quantitatively analyzed. The results showed that the inspection can be carried out to hold strength at a relatively low level within the range of 0.3t to 0.5t (t represents the average life of anechoic tile) [50], as shown in Figure 11c.

## 5. Summary and Outlook

### 5.1. Summary

Of note, material parameters and structural forms of acoustic coatings are the main factors affecting the acoustic stealth performance. Overall, material properties are affected by its structural form. From local structure to overall performance, design of acoustic coatings needs to grasp the key research content of each stage. Wang et al. classified the underwater sound absorption materials, introduced the corresponding sound absorption mechanism, and compared their advantages and disadvantages, as summarized in Table 2.

After a literature investigation, we find that current research still has some shortcomings:

(1) Although the local-structure design based on traditional acoustic theory is clear, its preparation is difficult, or the parameters of the prepared materials are contradictory to the setting parameters, which make it hard to achieve the expected performance [10].
(2) Some theoretical studies are limited in terms of preparation techniques, and they lack experimental verification. Examples are specified in Table A1 of Appendix A. Owing to its complicated structure, preparation of acoustic coating is difficult, which cannot be verified by experiments, thus making it more difficult for practical applications.
(3) Few researches focus on other performance requirements for acoustic coatings during design, such as temperature performance [52], inspection [53]. In different seas, depths, and seasons, seawater temperature changes significantly [1]. For more realistic underwater conditions, it is suggested to investigate mechanical and acoustic performances (such as complex elastic modulus) of elastomers in different temperatures and pressures [52].
(4) Researches on low-frequency sound absorption of acoustic coatings are few, especially the study of underwater acoustic materials below 1000 Hz.

### 5.2. Outlook

(1) Compared to passive control, the active control of sound waves is more adaptable and has broad application prospects. Accordingly, the design of active acoustic coatings will be a major direction for future development.
(2) Overall modeling and prediction of acoustic coatings under the coupling state of the shell structure require further study. When acoustic coatings are fabricated on the shell, acoustic characteristics in the coupling state of the structure and fluid are different from that in the natural state [54].
(3) Applications of other acoustic metamaterials in underwater environments are also worthy of further research, such as thin-film acoustic metamaterials [55,56], Helmholtz resonator type acoustic metamaterials [57], double-negative (negative effective density and negative bulk modulus) acoustic metamaterials [58], acoustic cloak [59], and so on.
(4) Theoretical breakthroughs have been brought by the electricity–mechanics–acoustics analogy [60] and mutual learning between acoustic methods and optical methods; structural design breakthrough brought by bionics [61]. Modern acoustics has a strong crossover and extensibility. This is especially true on the design of acoustic coatings. It involves various fields, such as mechanical engineering, material, chemical industry, etc., which requires communication and cooperation between scholars in different fields, and the strengthening of our own study in these fields.

However, it must be emphasized that there are still some key technical issues to be solved [45] as follows:

(1) The realization of low-frequency sound absorption performance of acoustic coatings without increasing thickness and weight of materials.
(2) The guarantee of strong sound absorption effect of acoustic coatings in low frequency and broadband range.
(3) The maintenance of sound absorption performance of acoustic coatings under deep-sea hydrostatic pressure.

## Figures and Tables

**Table 1:** Substrate materials of acoustic coatings [11].

Country	Acoustic Coating Substrates
Germany	Composite rubber
United States	Polyurethane, glass fiber, butyl rubber
Russia	Styrene-butadiene rubber, polybutadiene rubber, rubber ceramics
France	Polyurethane, polysulfide rubber
United Kingdom	Polyurethane
Japan	Neoprene
China	Styrene-butadiene rubber, polyurethane

**Table 2:** Classification of underwater sound absorption materials, main sound absorption mechanism, advantages and disadvantages [51].

Classification	Main Sound Absorption Mechanism	Advantages and Disadvantages
pure polymer material	viscous dissipation, heat transfer absorption, molecular relaxation absorption	excellent physical and chemical properties, easy to vulcanization molding, high viscoelasticity and excellent damping properties; large size, generally low strength, not resistant to hydrostatic pressure
particle-filled material	acoustic scattering, waveform transformation	improving sound absorption performance, improving the overall strength; large size, random distribution of the particle, its own characteristics are diluted by the matrix material
impedance-grading type material	viscoelastic internal friction, elastic relaxation	simple structure, good process molding, good sound absorption effect; large size, energy consumption mechanism is relatively simple
porous (foam) material	fluid vibration and friction in the hole	lightweight, high strength, resistant to hydrostatic pressure; bad low-frequency sound absorption performance, not resistant to seawater corrosion
cavity resonance type material	cavity resonance, waveform transformation, intrinsic properties of polymer material	better solving the problem of low-frequency sound absorption; low material strength, not resistant to hydrostatic pressure
phononic crystal	Bragg scattering, local resonance sound absorption	realizing control of long-wavelength acoustic waves by small-scale materials; regulation band is narrow, polymer matrix will cause bad sound absorption performance with the increase of hydrostatic pressure

## Appendix A

The appendix is to specify for some references the nature of the work.

**Table A1:** The nature of the work for some references.

References	Theoretical/ Numerical Work	Experimental Work	Year of Publication	References	Theoretical/ Numerical Work	Experimental Work	Year of Publication
15	√		2018	34	√		2019
16	√		2018	35	√		2015
17	√	√	2010	36	√		2019
18	√		2017	37	√		2019
19	√		2019	38	√		2017
20	√		2018	39	√		2019
21	√		2016	40	√		2017
22	√		2018	41	√	√	2015
23	√		2018	42	√	√	2019
24	√		2019	43	√	√	2014
25	√		2017	44	√	√	2017
26	√	√	2016	45	√		2013
27	√	√	2012	46	√		2014
30	√		2012	47	√		2016
31	√	√	2009	48	√	√	2017
32	√	√	2012	49	√	√	2018
33	√		2017	50	√		2018
